# Association between the LACE+ index and unplanned 30-day hospital readmissions in hospitalized patients with stroke

**DOI:** 10.3389/fneur.2022.963733

**Published:** 2022-10-05

**Authors:** Adalia H. Jun-O'Connell, Eliza Grigoriciuc, Brian Silver, Kimiyoshi J. Kobayashi, Marcey Osgood, Majaz Moonis, Nils Henninger

**Affiliations:** ^1^Departments of Neurology, University of Massachusetts Chan Medical School, Worcester, MA, United States; ^2^Departments of Internal Medicine, University of Massachusetts Chan Medical School, Worcester, MA, United States; ^3^Departments of Psychiatry, University of Massachusetts Chan Medical School, Worcester, MA, United States

**Keywords:** stroke readmission, LACE+ index, quality improvement, stroke readmission risk, 30-day hospital readmission prediction

## Abstract

**Background:**

The LACE+ index is used to predict unplanned 30-day hospital readmissions, but its utility to predict 30-day readmission in hospitalized patients with stroke is unknown.

**Methods:**

We retrospectively analyzed 1,657 consecutive patients presenting with ischemic or hemorrhagic strokes, included in an institutional stroke registry between January 2018 and August 2020. The primary outcome of interest was unplanned 30-day readmission for any reason after index hospitalization for stroke. The 30-day readmission risk was categorized by LACE+ index to high risk (≥78), medium-to-high risk (59–77), medium risk (29–58), and low risk (≤ 28). Kaplan-Meier analysis, Log rank test, and multivariable Cox regression analysis (with backward elimination) were used to determine whether the LACE+ score was an independent predictor for 30-day unplanned readmission.

**Results:**

The overall 30-day unplanned readmission rate was 11.7% (194/1,657). The median LACE+ score was higher in the 30-day readmission group compared to subjects that had no unplanned 30-day readmission [74 (IQR 67–79) vs. 70 (IQR 62–75); *p* < 0.001]. On Kaplan-Meier analysis, the high-risk group had the shortest 30-day readmission free survival time as compared to medium and medium-to-high risk groups (*p* < 0.01, each; statistically significant). On fully adjusted multivariable Cox-regression, the highest LACE+ risk category was independently associated with the unplanned 30-day readmission risk (per point: HR 1.67 95%CI 1.23–2.26, *p* = 0.001).

**Conclusion:**

Subjects in the high LACE+ index category had a significantly greater unplanned 30-day readmission risk after stroke as compared to lower LACE+ risk groups. This supports the validity of the LACE+ scoring system for predicting unplanned readmission in subjects with stroke. Future studies are warranted to determine whether LACE+ score-based risk stratification can be used to devise early interventions to mitigate the risk for unplanned readmission.

## Introduction

Unplanned hospital readmission following a stroke is common in the United States, with reported rates of 12–21% within 30 days, reaching up to 55% within 1 year (1–5) after the index event. Unplanned 30-day readmission has become an important quality measure in the United States (6–8), with the Centers for Medicare and Medicaid Services (CMS) connecting it with payment determination and penalties (8). Although many modifiable factors for stroke prevention are well recognized such as hypertension, diabetes mellitus, dyslipidemia and smoking (9), rates of unplanned readmission after a stroke are high (1). In part, unplanned readmissions are related to challenges surrounding the hospitalization, such as inadequate post-discharge support, insufficient follow-up, therapeutic errors, and failed hand offs (10). Indeed, most unplanned readmissions following a stroke are due to factors unrelated to recurrent stroke (5). Focusing solely on optimizing stroke risk factors may not reduce readmissions. Accordingly, identification of patients at high risk for readmission is essential and may afford an opportunity to mitigate precipitating factors, reducing the risk for unplanned 30-day readmission.

The LACE+ index is known as an extension of validated LACE index (length of stay, acuity of admission, co-morbidities, emergency department use within 6 months) which also includes other relevant factors (such as age, sex, and number of urgent admissions in previous year) (11). It has been introduced to identify patients at risk for unplanned 30-day readmission (11) and has been used in several medical conditions, including surgical patients and cancer (12–15). Advantage of this score is convenience in which it can be automatically computed by electronic medical record system, and most importantly, is a ubiquitous tool that can leverage the entire multi-disciplinary care team to help devise discharge strategies. Finally, it is known to be very accurate for predicting unplanned readmissions (16). Yet, its utility for predicting readmission after stroke is uncertain.

In this study, we sought to determine the association between the LACE+ index and 30-day unplanned readmission risk after hemorrhagic and ischemic stroke as the primary diagnosis in index admission. Our primary objective was to identify the overall incidence of 30-day unplanned readmission for any reason after index hospitalization for stroke and whether the LACE+ index was independently associated with unplanned readmission within 30-days of the index stroke.

## Methods

### Study cohort

We retrospectively analyzed prospectively accrued adult patients (greater than age 18 years) who were evaluated at the University of Massachusetts Memorial Medical Center (UMMMC) in Worcester, Massachusetts for an acute ischemic or hemorrhagic stroke between January 2018 and August 2020. The inpatient admission diagnosis of stroke was identified through the institution electronic medical record data as based on relevant ICD-9 and ICD-10 codes for the principal diagnosis. We excluded patients who died during the index admission or were discharged to hospice. The study was approved by our Institutional Review Board (IRB), and a Health Insurance Portability and Accountability Act (HIPPA) waiver of informed consent was approved. Our manuscript was prepared according to the Strengthening the Reporting of Observational Studies in Epidemiology guidelines (http://www.strobe-statement.org) (17).

All diagnoses were first established by the treating board-certified neurologist and confirmed by an abstracting physician (E.G.). Conflicting diagnoses were resolved by consensus after adjudication by a board-certified vascular neurologist (A.J.O.).

### Data collection

Patient demographics (race and ethnicity), insurance information, index admission length of stay (LOS), co-morbidities, preadmission medications, admission National Institutes of Health Stroke Scale (NIHSS), admission modified Rankin Score (mRS), discharge status, total admission cost in dollars, and LACE + score on discharge were collected for all patients by review of the medical records through the electronic medical record system. This captures information on patients admitted at institutions other than the University Campus, specifically UMMHC affiliated regional hospitals within Massachusetts. Two investigators independently reviewed all charts to confirm the qualifying index diagnosis as well as the outcome of interest.

### Definitions

The primary outcome of interest was the 30-day unplanned readmission, defined as a subsequent unplanned admission, occurring within 30-days of the discharge date from the index admission (18). The primary predictor of interest was the LACE+ index risk category assessed on the day of discharge. The LACE+ index was defined as a total score that consists of the variables of LACE index, including hospital length of stay (L), admission acuity (A), comorbid conditions *via Charlson Comorbidity Index*(C), emergency department utilization within 6 months before the admission (E), along with the sex, age, hospital teaching status, acute procedures and diagnoses in the index admission, and number of readmissions in the year before the index admission (11). The 30-day readmission risk was categorized by LACE+ scores: high risk (≥78), medium high (59–77), medium risk (29–58), and low risk (≤ 28) (11). The LACE+ scores were categorized to as to recognize different risk groups as our institution also uses these ranges for admissions to alert healthcare teams providing care. The index admission was defined as the admission of the starting point for studying repeat hospital visits (7). For subjects with more than one readmission during the 30-days after discharge only the first readmission affiliated within our medical record system was counted.

### Statistical analyses

Data are reported as median (interquartile range) unless otherwise stated. Univariate comparisons were performed with χ^2^, Fisher exact, Mann-Whitney U tests, and Kruskal Wallis ANOVA on Ranks as appropriate. A two-sided *p* < 0.05 was considered statistically significant in all analyses. To calculate corrected levels of significance in cases of multiple comparisons in the univariate analyses, adjusted significance level was calculated using Bonferroni correction. Kaplan-Meier analysis, Log rank test, and multivariable Cox regression analysis (with backward elimination) were used to determine whether the LACE+ index category was associated with 30-day readmission. We included the LACE+ index both as continuous and categorical variable in the Cox-regression models to calculate hazard ratios (HR) with corresponding 95% confidence intervals (CI). Models were adjusted for discharge status, index admission mRS, dyslipidemia, anti-platelet therapy use, and anticoagulation use. All statistical analyses were performed using IBM SPSS Statistics version 20.0.0 (IBM, Armonk, NY).

## Results

**Figure 1:**
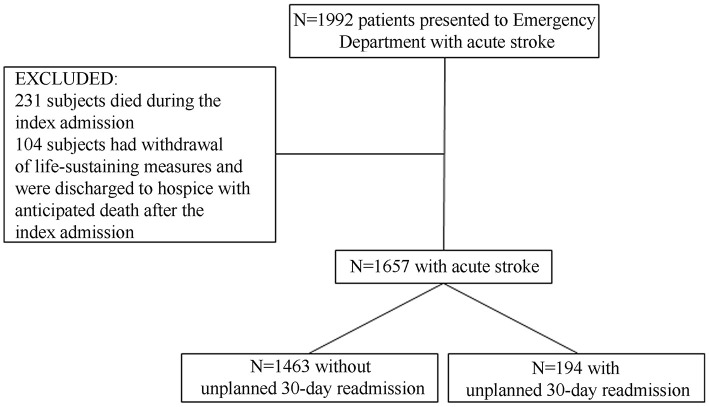
Patient flow chart.

Figure 1 depicts the flow chart of the study. We identified 1,992 acute stroke patients of whom 1,657 patients fulfilled the study criteria. Among included subjects 194 (11.7%) had an unplanned 30-day readmission [8 (4.1%) had more than 1 unplanned readmission].

### Factors associated with unplanned 30-day readmission

The baseline characteristics of the studied population as stratified by unplanned 30-day readmission vs. no readmission are shown in Table 1. Subjects with the unplanned 30-day readmissions were older [median 72 (IQR 62–82) vs. 68 (IQR 58–79) years; *p* = 0.014], had higher admission NIHSS [median 5 (IQR 2–13) vs. 4 (IQR 1–10); *p* = 0.007], higher pre-admission mRS [median 1 (IQR 0–2) vs. 0 (IQR 0–1); *p* < 0.001], and overall higher prevalence of pre-existing risk factors, including hypertension, diabetes mellitus, prior stroke/TIA's, atrial fibrillation, coronary artery disease, and heart failure as compared to the patients without unplanned readmission (*p* < 0.05, each). Moreover, readmitted patients had a longer length of stay [median 5 (IQR 3–11) vs. 4 (IQR 2–7) days; *p* < 0.001] and higher total cost of care associated with the index admission as compared to subjects without unplanned readmission [median $ 25,381 (IQR 13,283–46,667) vs. $ 18,210 (9,442–33,236); *p* < 0.001].

**Table 1:** Patient characteristics stratified by absence vs. presence of unplanned 30-day readmission.

**Characteristics**	**No unplanned readmission (*n* = 1,463)**	**30-day unplanned readmission** **(*n* = 194)**	***P*-value**
**Age** [Years; Median (IQR)]	68 (58–79)	72 (62–82)	0.014
**Gender**			0.540
Female	694 (47.4%)	87 (44.8%)	
Male	769 (52.6%)	107 (55.2%)	
**Race**			0.595
Asian	39 (2.7%)	5 (2.6%)	
Black	80 (5.5%)	13 (6.7%)	
Other	119 (8.1%)	20 (10.3%)	
White	1,216 (83.1%)	156 (80.4%)	
**Ethnicity**			0.248
Hispanic	131 (9.0%)	21 (10.8%)	
Non-hispanic	1,316 (90.0%)	173 (89.2%)	
Unknown	16 (1.1%)	0 (0.0%)	
**Insurance**			0.410
Medicare	843 (57.6%)	120 (61.9%)	
Medicaid	198 (13.5%)	17 (8.8%)	
Commercial	369 (25.2%)	49 (25.3%)	
Military	25 (1.7%)	5 (2.6%)	
Others	22 (1.5%)	3 (1.5%)	
Uninsured	6 (0.4%)	0 (0.0%)	
**Primary index admission diagnosis**			0.854
Ischemic	1,139 (77.9%)	150 (77.3%)	
Hemorrhagic	324 (22.1%)	44 (22.7%)	
Index admission NIHSS, Median (IQR)	4 (1–10)	5 (2–13)	0.007
Index admission mRS, Median (IQR)	0 (0–1)	1 (0–2)	< 0.001
**Pre-existing risk factors**			
Hypertension	1,085 (74.2%)	157 (80.9%)	0.043
Dyslipidemia	1,305 (89.2%)	149 (76.8%)	< 0.001
Diabetes	424 (29.0%)	77 (39.7%)	0.003
History of prior	354 (24.2%)	94 (48.5%)	< 0.001
Stroke or TIA			
Atrial fibrillation	369 (25.2%)	69 (35.6%)	0.003
Coronary artery disease	273 (18.7%)	70 (36.1%)	< 0.001
Congestive heart failure	176 (12%)	45 (23.2%)	< 0.001
**Index admission medications**			
Statin	692 (47.3%)	104 (53.6%)	0.108
Antihypertensive	934 (63.8%)	138 (71.1%)	0.046
Anti-glycemic	337 (23.0%)	42 (21.6%)	0.716
Antiplatelet	561 (38.3%)	93 (47.9%)	0.012
Oral anticoagulant	189 (12.9%)	41 (21.1%)	0.003
Total cost ($), Median (IQR)	18,179 (9,442–33,236)	25,381 (13,283–46,667)	< 0.001
Length of Stay, Median (IQR)	4 (2–7)	5 (3–11)	< 0.001
LACE+ score, Median (IQR)	70 (62–75)	74 (67–79)	< 0.001
**LACE+** **score risk category**			< 0.001
Medium risk (29–58)	162 (11.0%)	13 (6.7%)	
Medium-high-risk (59–77)	1,044 (71.4%)	114 (58.8%)	
High risk > 77	257 (17.6%)	67 (34.5%)	
**Discharge status**			< 0.001
Home	638 (43.6%)	47 (24.2%)	< 0.001
Short-term nursing facility	224 (15.3%)	50 (25.8%)	< 0.001
Inpatient rehabilitation	582 (39.8%)	97 (50.0%)	0.008
Other	19 (1.3%)	0 (0.0%)	0.154
**Discharge status to home**			0.023
Routine	444 (70.3%)	24 (51.1%)	
Home with services	172 (27.2%)	21 (44.7%)	
Left against medical advice	16 (2.5%)	2 (4.3%)	

Data are median (IQR) or n (%). IQR, Interquartile Range; NIHSS, National Institutes of Health Stroke Scale; mRS, Modified Rankin Score; TIA, Transient ischemic attack.

**Figure 2:**
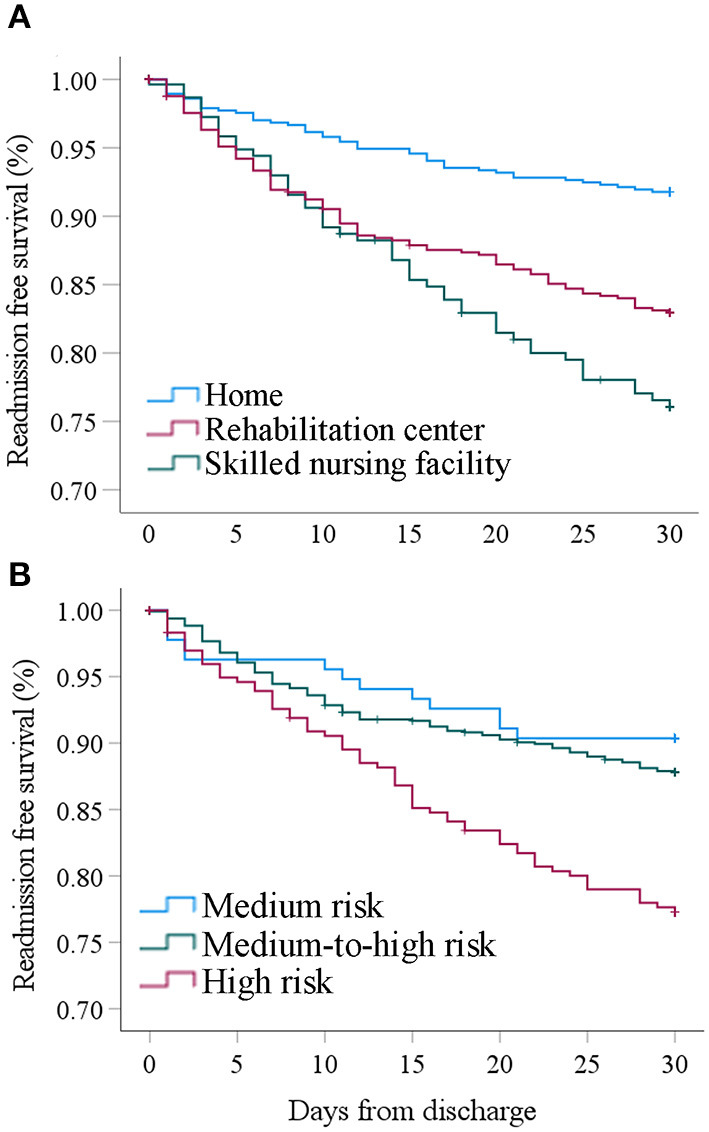
**(A)** Cumulative 30-day readmission free survival stratified by discharge category. **(B)** Cumulative 30-day readmission free survival stratified by LACE+ risk category.

We also found a significant association of the discharge disposition with risk of unplanned readmission. Readmitted subjects were less frequently discharged home (24.2 vs. 43.6%, *p* < 0.001) and more frequently discharged to an inpatient facility 75.8 vs. 55.1%; *p* < 0.05), and Kaplan-Meier analysis stratified by the discharge dispositions indicated that subjects discharged to a skilled nursing facility (SNF) had the greatest risk for unplanned readmission (Figure 2A). Subjects requiring a higher utilization of outpatient services such as visiting nurses had a greater unplanned 30-day readmission rate compared to subjects that were not readmitted (44.7 vs. 27.2%, *p* < 0.05).

### Causes for 30-day unplanned readmission

Information for the cause of readmission was available in 184 patients (94.8%): 64.1% of the readmissions were due to non-neurological diagnoses, whereas 35.9% were due to acute neurological complications. The three most utilized service lines included hospital medicine (48.4%), neurology (24.5%), and cardiology (7.6%). The most common causes that lead to readmission were infection (24.7%), recurrent strokes (21.0%), and cardiac complications such as atrial fibrillation and congestive heart failure exacerbation (17.2%).

### Association between LACE+ index and unplanned 30-day readmission

Compared to subjects without 30-day readmission, subjects that were readmitted within 30-days of discharge had a significantly higher LACE+ index score [74 (IQR 67–79) vs. 70 (IQR 62–75); *p* < 0.001]. None of the included subjects had a LACE+ index ≤ 28 (low risk category). Readmitted subjects were significantly more often categorized as high risk based on the LACE+ index (34.5 vs. 17.6%, *p* < 0.001) as shown on Table 1.

On Kaplan-Meier analysis, the cumulative 30-day readmission free survival was significantly lower for subjects with a high-risk LACE+ index as compared to subjects with medium-risk (*p* = 0.002) and medium-to-high risk (*p* < 0.001) groups. There was no difference in the readmission rate between subjects in the medium risk vs. medium-to-high risk LACE+ categories (*p* = 0.396) (Figure 2B).

On fully adjusted multivariable Cox-regression, a greater LACE+ risk score was independently associated with a higher 30-day readmission risk (per 10 points: HR 1.32, 95% CI 1.13–1.54, *p* < 0.001). Table 2 summarizes the results from the multivariable Cox regression analysis for factors associated with 30-day readmission. Factors independently associated with unplanned 30-day readmission included a high-risk LACE+ score, home discharge status, index admission mRS, dyslipidemia, antiplatelet use, and anticoagulation use (*p* < 0.05, each). Overall, subjects in the highest risk LACE+ category had a 62.5% higher probability (defined as HR divided by HR plus one) to be readmitted within 30-days of discharge as compared to subjects that were not high-risk (HR 1.667 95% CI 1.23–2.26, *p* = 0.001; Table 2).

**Table 2:** Variables associated with 30-day unplanned readmission on multivariable Cox-regression.

**Study variable**	**Hazard ratio (95% CI)**	***p*-value**
High risk LACE+ score	1.667 (1.229–2.260)	0.001
Home discharge status	0.527 (0.373–0.745)	< 0.001
Index admission mRS	1.238 (1.121–1.367)	< 0.001
Dyslipidemia	0.286 (0.200–0.409)	< 0.001
Antiplatelet use	1.439 (1.061–1.952)	0.019
Anticoagulation use	1.569 (1.098–2.242)	0.013

CI, confidence interval.

## Discussion

We have found that a higher LACE+ index assessed at the time of discharge is independently associated with unplanned readmission risk after stroke. This is an important finding as the predictive utility in stroke patients was previously uncertain, although the utility of the LACE+ index to predict the risk of unplanned hospital readmission has been previously validated in a large sample of medical and surgical patients (11). The need to better understand this issue is highlighted by the fact that stroke patients are at particularly high risk for unplanned 30-day readmission (1). In our cohort, 11.7% subjects had an unplanned readmission within 30 days, which is close to the previously reported rates that ranged from 12 to 21% (1).

In our study, patients with a high-risk LACE+ index (score ≥ 78) had a 62.5% greater probability of unplanned 30-day readmission than patients not considered high risk. This is an important finding, indicating that the LACE+ index may be used for stroke patients, creating an opportunity to identify patients at risk for preventable readmissions. This is important as unplanned readmission is a serious, costly issue in American healthcare system (19) for which identifying patients at high risk for readmission may potentially lead to an opportunity to address precipitating factors and create an opportunity to recognize issues surrounding the patient care. For example, identifications of readmitted patients led to interventions surrounding organized transition of care program, which have been shown to reduce 30-day readmissions in stroke patients (20). Furthermore, focus on clear goals of care surrounding code status and social engagement have been shown to have a positive impact on readmission reduction (21, 22). However, there needs more work and understanding to improve reduction in this patient safety and quality problem.

Most readmissions in stroke patients are due to non-neurological issues (14). Attention to potential stroke-specific complications, such as aspiration pneumonia and deep venous thrombosis represent viable targets for mitigating post-stroke complications and unplanned readmission. By utilization of LACE+ index in stroke at discharge, one could consider re-allocation of resources toward higher risk patients to implement individualized transition care plans with close follow-up focused on prevention and early identification of conditions that increase the risk for readmission (23). Further studies will be required to determine specific implementation strategies in at-risk patients (24).

Interestingly, it was previously reported that readmission risk was highest in patients that were directly discharged home as compared to acute facilities (25, 26). However, we found that discharge to home after stroke was independently associated with a lower unplanned readmission risk. A likely reason for this observation is that these patients have a lower medical complexity and better-preserved functional status than patients that were discharged to facilities providing a higher level of care (27). Consistent with this notion, we found that patients with unplanned 30-day readmission after initial home discharge had a greater utilization of home services. This may offer an opportunity to optimize transition services in patients deemed stable for home-discharge.

Limitations of our study include its retrospective study design, which may have introduced bias. Second, the study population was obtained from a single tertiary care center, hence may not be representative of population samples and limiting generalizability and may not be appropriate for evaluating the outcomes in other patient populations. Nevertheless, the electronic medical record system allowed the review of medical records outside our study institution. Our observed readmission rates are in line with previously reported rates that ranged from 12 to 21% (1) indicating that our results likely translate to other hospital settings. Nevertheless, our results need to be interpreted with caution as it may not have captured hospitalizations that are not associated with the shared electronic medical record system. Third, the data were obtained from the institution diagnoses codes. However, we performed two-person chart reviews to confirm the correct diagnoses codes.

## Conclusion

A high LACE+ index category was independently associated with a greater unplanned 30-day readmission after stroke, highlighting its potential utility for predicting unplanned readmission in subjects with stroke. Future studies are warranted to determine whether LACE+ score-based risk stratification can be used to devise early interventions to mitigate the risk for unplanned readmission in stroke patients as to improve patient outcome and quality care, and to understand if resource allocation toward higher LACE+ index stroke survivors have better patient outcomes.

## Data availability statement

The raw data supporting the conclusions of this article will be made available on reasonable request. Access requests should be directed to the corresponding author(s).

## Ethics statement

The studies involving human participants were reviewed and approved by UMass Chan Medical School Institutional Review Board. Written informed consent for participation was not required for this study in accordance with the national legislation and the institutional requirements.

## Author contributions

AJ-O and EG: data acquisition. AJ-O, EG, and NH: interpretation of data. AJ-O and NH: study concept and design and drafting of the manuscript. EG, BS, KK, MM, and MO: critical revision of the manuscript for important intellectual content. NH: statistical analysis. All authors contributed to the article and approved the submitted version.

## Conflict of interest

Author BS received compensation for review of medico legal malpractice cases, for adjudication of stroke outcomes in the Women's Health Initiative, and authorship for Ebix Medlink, Medscape. Author NH was supported by W81XWH-19-PRARP-RPA from the Department of Defense Congressionally Directed Medical Research Programs (CDMRP). Author AJ-O received compensation for adjudication of stroke outcomes in the Women's Health Initiative. The remaining authors declare that the research was conducted in the absence of any commercial or financial relationships that could be construed as a potential conflict of interest.

## Publisher's note

All claims expressed in this article are solely those of the authors and do not necessarily represent those of their affiliated organizations, or those of the publisher, the editors and the reviewers. Any product that may be evaluated in this article, or claim that may be made by its manufacturer, is not guaranteed or endorsed by the publisher.
